# Reply

**DOI:** 10.1016/j.gastha.2026.100890

**Published:** 2026-01-31

**Authors:** Marcia Irene Canto, Elizabeth Abou-Diwan, Helena Saba, Amanda L. Blackford, Michael Goggins

**Affiliations:** Division of Gastroenterology and Hepatology, Department of Medicine, Johns Hopkins University School of Medicine, Baltimore, Maryland

We appreciate your comment regarding the significance of a dilated main pancreatic duct (MPD) in patients with chronic pancreatitis, particularly in patients with hereditary chronic pancreatitis who have an elevated risk for pancreatic cancer.

We do not see very many patients with hereditary chronic pancreatitis in our Cancer of the Pancreas Screening Study consortium. In the cohort study published recently (Diwan et al, Gastro Hep Adv. 2025 PMID: 41142531), we had only 1 patient who had a PRSS1 mutation and a family history of pancreatic cancer. This high-risk individual presented with severe chronic calcific pancreatitis as might be expected and a pancreatic cyst, which proved to be a low-grade branch duct intraductal papillary mucinous neoplasm.

We do agree, in theory, that the significance of a dilated MPD in hereditary chronic pancreatitis patients may be less predictive for malignant transformation. The predictive value of mild MPD dilatation should be interpreted within the scope of the studied Cancer of the Pancreas Screening Study population as described and may not be generalizable to patients with hereditary chronic pancreatitis. Clearly, more data are needed from pancreatic cancer surveillance centers with a larger number of these patients to determine other predictors of elevated risk for neoplastic progression.

